# Sailing Across the Atlantic: An Exploration of the Psychological Experience Using Arts-Based Research

**DOI:** 10.3389/fpsyg.2020.572028

**Published:** 2020-10-14

**Authors:** Anita Pipere, Kristīne Mārtinsone, Laura Regzdiņa-Pelēķe, Ingūna Grišķeviča

**Affiliations:** Department of Health Psychology and Pedagogy, Riga Stradiņš University, Riga, Latvia

**Keywords:** arts-based methods, diary, freehand drawings, adventure tourism, psychological experience, transatlantic sailing

## Abstract

The reported study pursues the bidimensional objective, namely, (1) to grasp the essence of the psychological experience of a transatlantic journey and (2) to observe the potential of arts-based research for the enhancement of the well-being of sailors. The sample consisted of three males and one female – Latvian citizens, aged 36–68 years, with higher education and different sailing experience. In the context of a case study grounded on arts-based research methods, the freehand drawings of self-portraits or something important during the day were obtained using individual diaries containing daily reports on the sailing experience. After the journey, individual drawings prompted interviews organized for each participant. The results were triangulated with data from the logbook. In their drawings, participants focused more on the environment rather than on self-representation, that is, telling the story about “here and now” in terms of specific time (journey), place (boat), and their relationship with nature, as well as dealing with problems during the trip. The psychological experience of transoceanic sailing matched the three general dimensions (dynamics, context, and content of experience) of the conceptual framework constructed for this study. The three dimensions of experience were equivalently represented in research data; however, only dynamics and context were fully delineated in all theoretical subcategories. As to the potential of arts-based research for the enhancement of sailors’ well-being, the findings show both direct and indirect evidence concerning their psychological and existential well-being. Further research is still needed to confirm the findings on a broader scale and in other contexts.

## Introduction

With the rapid growth of the profit-oriented global tourism industry (Ruhanen and Shakeela, 2013), there is an increasing interest in adventure tourism. This trend offers different and much more extreme nature of experience in comparison with traditional commercial tourism. Transoceanic sailing can be viewed not only as the “hard” adventure tourism (Plog, 1991), but also the extreme case of “blue care” – use of outdoor surface waters for therapeutic interventions to promote positive changes and boost health and well-being (Schijf et al., 2017; Britton et al., 2018). Studying such extraordinary experiences could also provide a glimpse of the future of travel (Laing and Crouch, 2005). The recent advance of psychological research on sailing experience does not apply to transoceanic sailing, which is still in small numbers (Weston et al., 2009; Kjærgaard et al., 2015; Jirásek and Hurych, 2019). Exploration of transoceanic sailing could provide insight into contextually similar situations encountered during, for instance, COVID-19 pandemics (Ho et al., 2020), connected with long-term isolation in confined space (weeks long sailing trip) in generally uncertain and threatening conditions (sailing in the open ocean). Besides, scholars have already underlined the visual reflection of reality as fundamental to tourism and have called for the application of innovative visual methods, thereby enabling access to layers of meaning beyond the reach of more traditional qualitative methods (Feighery, 2003; Pritchard and Morgan, 2003; Rakić and Chambers, 2010). However, the real potential of such methods in tourism research is still to be discovered, and we will take the first steps in this direction, employing one of the dominant approaches within the paradigm of art-based research – using art processes (namely, free drawing) as tools to collect and present data to study a certain issue (Greenwood, 2012). The idea of researching human experience through the arts, first defined by McNiff (1989), in the given study, binds together art and tourism research focusing on the methodological complexities of the proposed research topic and the transformative nature of art-based research (McNiff, 2008) in terms of improving the well-being of research participants.

One of the main aims of tourism is to offer a reflective space for self-insight and examination of life priorities (Brown, 2013). The specific type of adventure tourism envisages voluntary engagement with increased risk or uncertainty, demanding physical activities, interacting with the natural environment to reach distant localities with natural destinations (Elsrud, 2001; Weber, 2001; Varley, 2006; Giddy and Webb, 2018). This form of tourism demands giving up control of the situation, living on the edge of chaos, thus allowing for skill development, personal growth, self-expression, and self-actualization (Haegeli and Pröbstl-Haider, 2016; Jirásek and Hurych, 2019).

In literature, the phenomenon of sailing is divided into boating tourism (i.e., ecotourism), cruise tourism, and transoceanic sailing (in our case – transatlantic sailing). Transoceanic travel, associated with Dionysian aspects of adventure, is different from ecotourism or mass experiences of cruise tourism (Jirásek and Hurych, 2019). Our interest in this article was not the transoceanic solo sailing or sail training, but rather sailing on a catamaran sailboat with five crew members and two passengers (from Gran Canaria to Cape Verde) on board. The transatlantic voyage on the Lagoon 45 catamaran sailboat Belle from Gran Canaria (Spain) *via* Cape Verde to Tobago (Trinidad and Tobago) lasted 23 days (from November 7 to December 2, 2019). The journey covered 2,980 nautical miles (5,518 km) and crossed five time zones. The temperature ranged between 20 and 30°C, waves (swells) varied from 1 to 4 m, and the wind varied from 10 to 30 knots (5–15 m/s). Life on board was divided into 4-h watches (odd jobs running the ship and its daily operations) and time off (reading, writing, recreation, and meals).

### Conceptual Framework for Psychological Experience of Sailing

In this study, “psychological experience” is defined in an extended way as “a category of thinking, a minimal unit of analysis that includes people (their intellectual, affective, and practical characteristics), their material and social environment, their transactional relations (mutual effects on each other), and affect” (Roth and Jornet, 2013, p. 107). Thus, the experience of transoceanic sailing reflects the mutual interdependence of environment and person, and therefore, the same environment will influence the continuous flux of experience (development) of every crew member and passenger in very different ways (Roth and Jornet, 2013).

A conceptual framework for the psychological experience of sailing was created to provide the deductive schema for the data analysis. This framework was not created as a hierarchical system but, using an integrative eclectic approach, conceded the complementing nature of different schools, theoretical approaches, and trends (Mārtinsone, 2016). Meanwhile, the systematic eclecticism (a subtype of integrative eclecticism) accommodated the selection of the most relevant components of existing theories and research outcomes for the specific research problem and methodological architecture (Norcross and Goldfried, 2005; Dattilio and Norcross, 2006). The inspection of theoretical, review articles, and qualitative and quantitative studies focused on (a) experience of transoceanic sailing (Kuhn, 2001; Jirásek, 2015; Jirásek and Hurych, 2019), (b) the connection between nature and well-being (Yalom, 1980; Wong, 2009; Passmore and Howell, 2014), and (c) transformative tourism experience (Kirillova et al., 2017a,b).

The constructed framework included three general dimensions – dynamics, context, and content of experience, namely, the temporal features (the process of the journey), spatial context (sailboat crossing the ocean), and psychological underpinnings of the journey. To elaborate, the dynamics of experience was conceived as the time dimension incorporating the process of the journey, eventually starting long before the actual journey, engagement in activities “here and now,” lack of essential reflection on existential given, episodes of sailing encouraging deeper reflection, and vision of self and world at the end of travel (Kirillova et al., 2017a,b). Spatial or environmental context emanated in the categories of natural beauty, the encounter with wildlife, and social connections among the crew (Jirásek and Hurych, 2019). The content of psychological experience was constructed using systematic eclecticism – integrating existentialism, existential humanism, and humanistic/transpersonal psychology. Elicited from the literature review, the categories of the meaning of life, spiritual transcendence (connection with something grand), and authentic mode of existence can be connected with the strand of existentialism (Kirillova et al., 2017a,b; Jirásek and Hurych, 2019); the categories of isolation, freedom, and death relate to the existential psychology (Yalom, 1980; Passmore and Howell, 2014), whereas self-actualization, self-representation (identity), and happiness (different aspects of well-being) represent the integration of positive psychology and humanistic/transpersonal psychology (Kuhn, 2001; Passmore and Howell, 2014).

### Arts-Based Research and Transatlantic Sailing

Since the psychological experience of transoceanic sailing is associated with multifaceted aspects difficult to measure, it was decided to use arts-based research that can provide a multilayered understanding of the phenomenon and offer a creative way to construct knowledge (Rydzik et al., 2013). Arts-based research is vigorously defended by many health professionals as a tool to move beyond text and is recommended as part of a multimethod interdisciplinary approach (Rees, 2018). Using freehand drawings or rich pictures (Cristancho et al., 2015) as data can promote the participants’ reflection on the complexity of the situation and provide insight into the hidden aspects of the research problem using the subconscious or unrealized feelings and perspectives (Rattine-Flaherty and Singhal, 2007; Cristancho, 2015; Cristancho et al., 2015). Thus, in terms of the therapeutic dimension of arts-based research, this approach can offer a way of communicating other than speech while bringing an opportunity to enhance psychological well-being. Offering time and space for reflection, active conceptualization, and contemplation (Gauntlett, 2007), the method of freehand drawing creates the conditions for improvement of existential health (Bagnoli, 2009). Also, using nonmechanical media as drawing, content is not limited by form, and depiction of the physical and abstract realities is unbounded (Banks, 2001).

To summarize, it can be assumed that psychological experience of transoceanic sailing, in general, would match the conceptual framework constructed for this study, while arts-based research could be helpful in the elicitation of reflection and contemplation, thus enhancing the existential health and well-being of sailors. The authors aim to describe the qualitative study on the psychological experience of transoceanic travel and explore the therapeutic and methodological potential of arts-based research for the enhancement of the well-being of sailors. Accordingly, the following research questions were set:

1. What is the essence of the psychological experience of sailing across the Atlantic revealed by arts-based research?
2. What is the potential of arts-based research for the enhancement of sailors’ well-being?

## Materials and Methods

### Research Design

The methodological framework for this study has been constructed as a case study (Yin, 2014) using arts-based research methods and viewing the event of a sailing journey across the Atlantic Ocean as a case (Hancock and Algozzine, 2006). The crew on the boat during the journey from its beginning to the end could be approached as a limited, integrated system – humans in their environment. The specific context of relationships between the experience of the crew from one side and limited space and time on the boat from the other side blurs the borders between the phenomenon (sailing experience) and its context (boat, crew, journey, ocean, etc.). The explored event can be viewed as a single case with four embedded units (Yin, 2014), i.e., the experience of four crew members – research participants. A qualitative case study was implemented as the most appropriate for the research questions related to the long experience in rare conditions (Vieira et al., 2017). As in the instrumental case study (Stake, 1995), the investigation uses the deductive approach to the theory development, assessing the alignment of the above described conceptual framework with the obtained data.

To enhance the trustworthiness in terms of data quality and variability, the given study aggregated and triangulated the drawings of participants as the second-degree indirect method where the researcher does not interact with the participant during the data collection, semistructured graphic elicitation interviews as a first-degree data – the researcher is in direct contact with the subjects and collect data in real time, and the analysis of logbook as the third-degree data source – independent analysis of already available artifacts (Lethbridge et al., 2005). As a part of a larger research project, the given article will focus only on the methods directly related to arts-based research.

### Participants and Ethics

Given the specific nature of this case study, the purposive sample consisted of crew members of sailboat as a closed system in a bounded context (Miles and Huberman, 1994), i.e., four Latvian citizens with higher education: participant A – a 50-year-old woman with 15-year-long sailing experience, participant B – a 68-year-old man with 44-year-long sailing experience; participant C – 50-year-old man with 30-year-long sailing experience; participant D – 36-year-old man with a short experience of recreational sailing. Only participant B had an experience of several transoceanic sailing trips; for other participants, it was first transoceanic sailing. Recruitment of participants was conducted in line with the modified snowball approach (Noy, 2006): the research idea was shared with one crew member who spread this invitation to other crew members. Of five crew members, four granted their participation in the study, signing the informed consent letters. Because of the sensitive ethical background, the research participants were asked to provide the feedback on article, thus boosting the research rigor utilizing external verification of research findings (Morse et al., 2002).

### Data Collection

#### Freehand Drawings (Travel Diary)

In this arts-based research, art processes were used both for collecting data and understanding the meaning within the drawings (McNiff, 2008; Greenwood, 2012), therefore providing fresh approaches and different perspectives on the studied issue (McNiff, 2013b; Dunn and Mellor, 2017). Drawings encouraged the participants to reflect in real time (here and now) and thus demonstrated the psychological experience of sailing, implicitly illustrating the potential of arts-based research. Because the interviews conducted by researchers were not possible in real time (during sailing), a series of drawings in this unique situation (transatlantic sailing) allowed access to the diversity of participants’ experiences that could not be accessed only by their written expressions (Dunn and Mellor, 2017). The given method also relates to the idea of “time-series analysis” (Jebb et al., 2015), when researchers analyze repeated observations on a single individual at regular intervals. Repeated observations were collected through a travel diary survey consisting of the same six tasks for each day. Besides the verbal tasks – answers on open or closed questions, the participants fulfilled the arts-based assignment instruction: “Please draw yourself today or your day and provide the title for your drawing.” Participants did not express any objections toward the drawing task during the instruction session before the journey.

#### Graphic-Elicitation Interviews

This method provided the context for reflection on the experience after the event and, similarly as the drawing method, enriched the researchers’ understanding of the participants’ psychological experience of sailing and the therapeutic potential of arts-based research. The posttravel semistructured interviews confronted participants with the series of their drawings. The modified approach for the visual data analysis (Bagnoli, 2001, 2009) determined the set of questions regarding each drawing: “Please describe what experience (memories) of the sailing journey can be associated with this drawing? What is the meaning of this experience? In what way does this drawing illustrate this meaning?” Finally, the participants were asked about their overall experience of participation in the given study, and which research methods had the largest impact on them. Also, it was asked if this arts-based research was beneficial (or disturbing) for their well-being during the journey and afterward. The interviews were audio recorded and later transcribed verbatim.

#### Document Analysis

The sailing logbook entries were used as the primary documents to be checked for specific deviations from normal sailing conditions. This analysis allowed the researchers to compare the objective situation during the day with the drawings and their posttravel explanations by the research participants.

### Data Analysis

Researchers analyzed drawings, their titles, and explanations by the authors as the single units of analysis, combining qualitative content analysis (Graneheim and Lundman, 2004) with a frequency analysis appropriate for a large number of obtained drawings. This nuance of quantification is much less encountered in arts-based research (McNiff, 1998, 2013a; Kara, 2015), although it seems quite relevant considering the selected methodology and research topic. The quality of the drawings was not taken into account during the data analysis. The coding frame for the deductive qualitative content analysis was based on the conceptual framework including main categories of dynamics, context, and content of sailing experience and their subcategories. Data were reviewed and coded for each participant first and then across all the participants to check for overlap and convergence of content and/or themes (Ayres et al., 2003). The four-stage triangulation of objects and subjects was used as a framework for data analysis: (1) the participants make an interpretation of their drawings; (2) researchers (individually and collaboratively) interpret the drawings and the data from graphic-elicitation interviews; (3) researchers compare the drawings and interview data with the entries of the logbook; (4) triangulation between the participants’ interpretation of drawings during the interviews, coordinated interpretation of drawings and interviews by three researchers, and entries of the logbook.

## Results

### The Psychological Experience of Transatlantic Sailing

Analyzing in total 93 drawings, their titles, and drawing-elicited interviews, it appeared that the travel diary of A contains 26 drawings (4 self-portraits, 1 picture of the crew, 21 pictures of surroundings), B has created 38 drawings (5 self-portraits, 1 portrait of the crew member, 32 pictures of surroundings), and C provided 23 drawings (13 self-portraits, 2 drawings with crew members, 8 pictures of surroundings), whereas D has created 6 drawings (4 self-portraits, 2 pictures of surroundings).

The preliminary qualitative and quantitative analyses of drawings (without the title and explanation by the authors) show that the largest and the most diverse group of themes pertains to the recreational and household activities (drinking, eating, calling home, reading the book, playing guitar, playing dice, diving, drying the pants). The next largest group represents the nature-related dimension (huge dorado, moon upside down, fluorescent plankton, dolphins, flying fish). The much smaller group reflects the issues such as trouble with a satellite phone and ghost nets (fishing nets lost in the ocean). Some pictures were related to the destination of the trip, a transcendent view of the journey, a distant view on the boat, and self-portraits with positive emotions.

**Figure 1:**
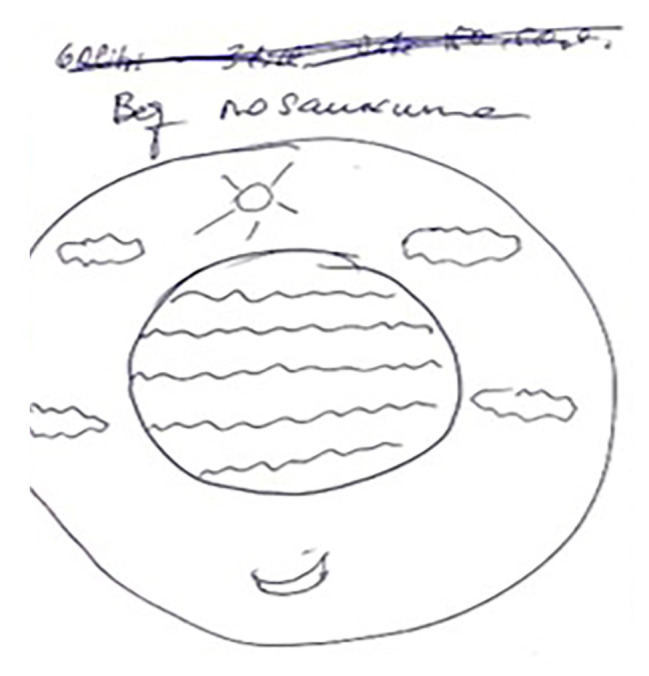
Spiritual transcendence (“no title”; participant A).

Next, we will compare the categories elicited from the specific content of drawings, their titles, and interviews with the conceptual framework of sailing experience in terms of each participant. Thus, dominant subcategories of the conceptual framework for A were existential experience, relationships with nature, spiritual transcendence (Figure 1), and space/time dimension. All dominant categories as well as the spending time onboard (here and now), social relationships, and happiness were evenly distributed during the journey, except for the emphasis on physical well-being and life/death categories in the first part of the trip.

**Figure 2:**
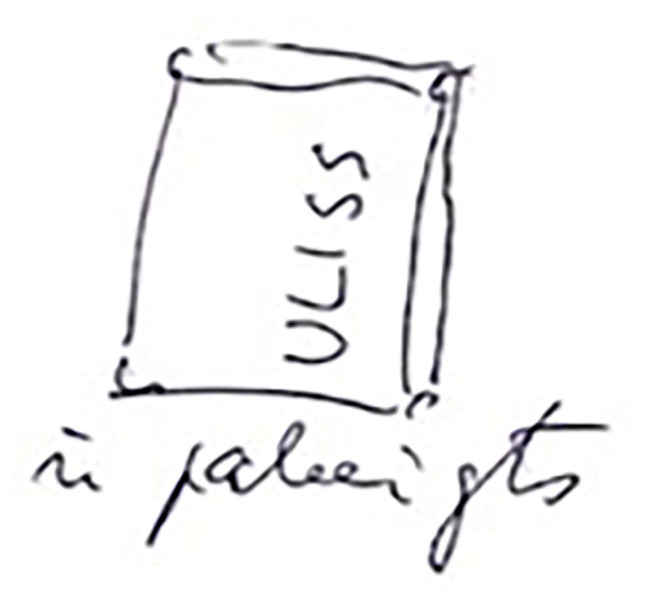
Spending time on the boat (“Ulysses – finished”; participant B).

For B, dominant subcategories were happiness, spending time on the boat (Figure 2) and entering port (here and now), and relationships with nature. In terms of dynamics, all main categories, as well as self-actualization and social relationships, were rather evenly distributed during the journey; only the technical details of sailing appear more in the first part of the journey.

**Figure 3:**
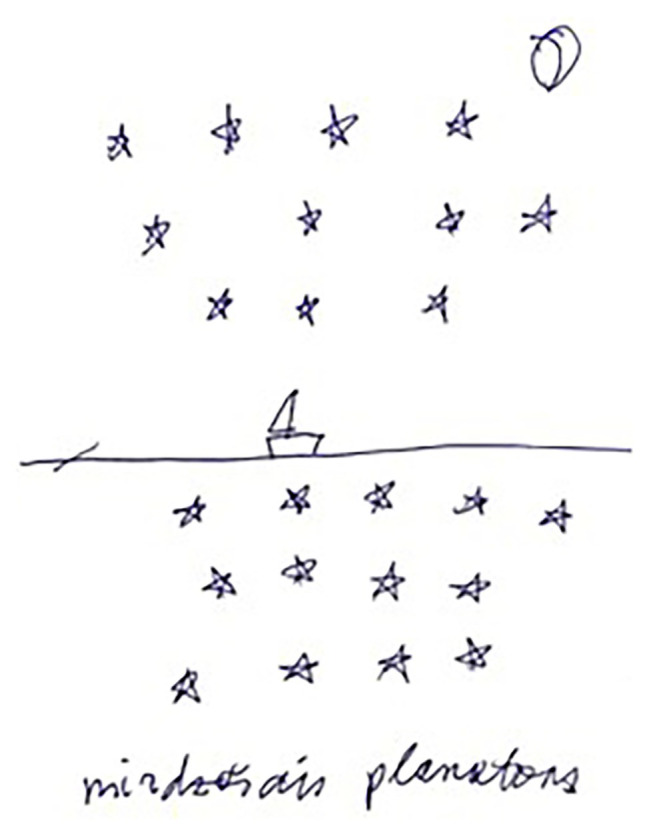
Relationships with nature (“fluorescent plankton”; participant C).

For C, subcategories such as relationships with nature (Figure 3), happiness, and spending time (here and now) prevailed. These categories along with the category of social relationships were evenly distributed during the time of travel; other categories emerged randomly in small numbers.

**Figure 4:**
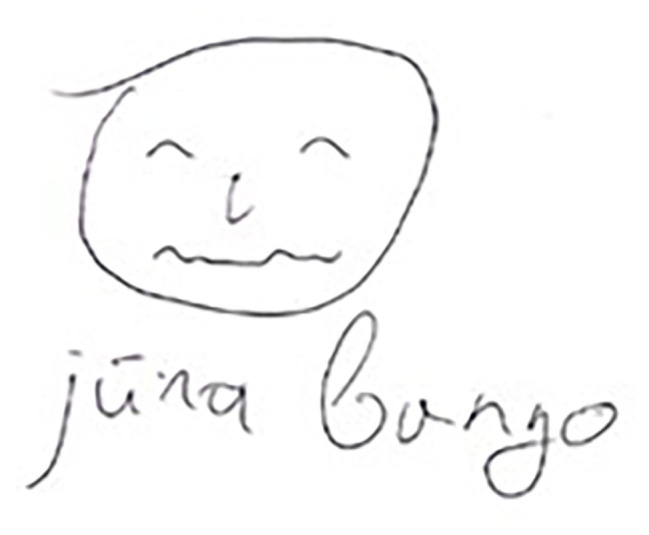
Physical well-being (“stormy sea”; participant D).

Six drawings by D depict the subcategory of physical well-being (Figure 4) and relationships with nature; besides, the drawings featuring physical well-being were dispensed across the whole series of drawings.

Now we will evaluate the alignment of the categories discerned from the specific drawings, their titles, and posttravel interviews for the whole sample with the (1) preliminary analysis of drawings, (2) individual dynamics of categories, and (3) categories of the conceptual framework for the sailing experience, focusing on three main categories: dynamics, context, and content of experience. Each unit of integral data, depending on the context, was coded in one to five categories; thus, the percentage of categories adds up to more than 100%.

#### Dynamics

In terms of the dynamics of experience – the time dimension incorporating the process of the journey, the preliminary view on the drawings affirms the prevalence of specific activities as well as the small number of drawings on existential issues as expected. Only some drawings (especially by A and D) at the end of the journey pertain to the deeper reflection and the vision of self and the world. In terms of the conceptual framework, it appears that sailing as a process (in dynamics) was represented in 80.6% of drawings, including time dimension (16%) and activities here and now (84%) such as spending time/household (35%), space/place dimension (25.4%), physical well-being (23.8%), technical details of sailing (11.1%), and boat (4.8%).

#### Context

Categories of spatial or environmental context such as natural beauty, the encounter with wildlife, and social connections were also widely represented in the integral data. The preliminary analysis shows the large group of nature-related drawings, while the social connections were represented mostly in an indirect way. For all samples, relationships with nature were among the dominant categories (especially for C). Comparing the data with the conceptual framework, it occurs that the context of sailing experience (79.6% of drawings) perfectly aligned with three theoretical subcategories – encounter with wildlife (51.4%), social connections (33.8%), and natural beauty (14.9%). The social connections were coded into the categories of relationships with crew members, connection with home, and belonging to the crew.

#### Content

The preliminary view identified fewer drawings representing the psychological content of experience. Participant A presented the largest number of drawings in this major category. In general, the number of drawings in the category of content was almost identical to the categories of dynamics and context (78.5, 79.6, and 80.6%, respectively). Making a comparison of the integral data with the conceptual framework, only subcategory, fully represented in integral data, was connected with positive psychology and humanistic/transpersonal psychology (74%), namely, self-actualization (18.5%), self-representation (identity; 13%), and happiness (different aspects of well-being; 68.5%). In terms of existentialism (19.2%), only spiritual transcendence was observed in the integral data, excluding the meaning of life and authentic mode of existence. Considering the existential psychology (6.8%), categories of isolation and freedom were missing, while the category of death was paired with the category of life.

Detailed exploration of entries in the sailing logbook and their comparison with 93 units of the analysis show that drawings and their interpretation at large matched the objective situation during the travel.

### Arts-Based Research and Sailors’ Well-Being

Because of the lack of arts-based research on sailing experience, the inductive approach to the data analysis related to the second research question was applied. The presentation will be structured around the evaluation of the study, specific research methods, and impact on participants’ well-being.

In respect to the whole research, participants admitted that although filling in the diary was a little restrictive, it had several positive outcomes for psychological well-being, such as satisfaction for realizing the commitment, good and interesting experience, dealing with urgent life questions, or “observation and reflection on daily experience, making this experience of adventure tourism richer – without such reflection my emotions and adventures would be just a nonspecific flow of memories, preserved only in some photos” (Participant A).

In terms of specific research methods, drawings and drawing-elicited interviews were recognized as the most interesting methods and the most powerful tools for the reflection and recycling of experience, with the potential to incite the existential well-being of sailors. According to B, “to figure out what to draw daily was the most interesting part. It never took longer than half a minute; thus, topics of drawings were impulsive.” Participant A compares drawings with mysterious symbols understandable just for the creator: “each drawing is a symbol for the whole experience, recalling or reviewing the drawing you can bring back the essence of this experience in all its splendor with sounds, smells, tastes, lights, pleasures and fears, insights and reflections.”

However, direct questions about the enhancement of well-being by arts-based research led to diverse answers starting from a relaxed perspective to openly positive self-questioning. B and D displayed the neutral attitude toward the drawings, more like an obligation to be met. Participant C demonstrated the ambivalent stance toward the arts-based research – drawing was comfortable for him, while the analysis of drawings was rather painful. He admitted that “daily drawings were spontaneous visual expressions, not the illustrations of psychoanalytical reflections.” Speaking about well-being, he suggests that reviewing drawings after the journey helped to recall the travel experience, thus encouraging delightful feelings. Finally, participant A wonders: “Would it be possible that this study has enhanced my psychological well-being enabling me to discover the power of drawing?”

## Discussion and Conclusions

This qualitative case study provided the short insight into the psychological experience of the transoceanic journey and the arts-based research as an environment for the possible enhancement of the well-being of sailors, as well as the methodological tool for such a specific study. In their drawings, participants focused more on the environment than on self-representation, while the specific content of the drawings tells the story about “here and now” in terms of specific time (journey) and place (boat) and relationships with nature, dealing with some problems during the trip. Fewer numbers of drawings represented the boat/journey and emotional state of participants.

The psychological experience of transoceanic sailing matched the conceptual framework constructed for this study. Three general dimensions – dynamics, context, and content of experience were equivalently represented in research data; however, only dynamics and context were fully represented in all theoretical subcategories. The content of psychological experience was matching only in terms of positive psychology and humanistic/transpersonal psychology, where the category of happiness took the dominant position. Theoretical categories such as the meaning of life, authentic mode of existence, isolation, and freedom were missing in data, while the category of death was complemented with the category of life. It seems that sailors mostly engage in a deeper reflection on sailing experience before or after the journey, while in the present time (on the boat) they mostly go for specific recall of daily events, depicted in drawings; thus, art becomes an illustration of “here and now” resembling the documental photograph. The findings echo the idea about tourism not only as an escape route but also means for self-discovery, actualization, and transcendence (Comic and Beograd, 1989). Although not all tourists all of the time are looking for existential well-being (Steiner and Reisinger, 2006), the search for meaning, connection, and self-actualization are important to many (Wang, 1999). Focusing on sociodemographic features, transcendence was more characteristic for the female sailor, while the older male sailor with the largest sailing experience generally depicted the moments of well-being and “household activities.” It should be noticed that relationships with nature were among the most frequent categories for all research participants.

As to the potential of arts-based research for the enhancement of sailors’ well-being, the findings show both direct and indirect evidence with respect to the psychological and existential well-being; however, some controversial trends in data demand further research with larger and more homogeneous samples to obtain a more convincing picture.

Speaking about the strength of this study, we should notice that “seeking participants’ interpretation of their drawings is invaluable as their visual product is highly subjective and can be easily misunderstood from a third perspective” (Cheung et al., 2016, p. 641). Three more advantages relate to the feedback from the research participants and triangulation of different data sources, as well as objects and subjects for the data analysis. Limitations of the study can be related to the small and heterogeneous sample that could hinder the transferability of findings and the short list of interview questions related to the reflection on arts-based research. However, the small, while purposive samples (Patton, 2002; van Rijnsoever, 2017) are the specific and valuable feature of any qualitative research (Sandelowski, 1996); besides, the transferability to other comparable contexts and situations (such as COVID-19 mentioned at the beginning of the article) in the presented research can be ensured by the detailed description of the research context. Similar weakness has been reported in other studies on sailing and extreme sports (Kuhn, 2001; Guthrie, 2008; Jirásek and Hurych, 2019; Reid and Kampman, 2020). Although the quality of drawings or drawing skills of participants were not considered in this study, the observation about the limited number of self-portraits may reflect the lack of confidence to use that form of expression.

To conclude, this inquiry has presented the detailed documentation of the innovative usage of arts-based research method in a new domain of psychological research – transatlantic sailing – and proves the multifaceted benefits of this method both as a tool for scientific inquiry and, to some extent, as the means of enhancing the well-being of participants of extreme adventure. In terms of practical application and further studies, this article suggests a novel approach to the studies of sailing experience propelling future exploration in the context of adventure tourism. The advanced methodology of arts-based research can be implemented in this field and other subfields of tourism, as well as research focusing on the enhancement of well-being and transformation.

## Data Availability Statement

The raw data supporting the conclusions of this article will be made available by the authors, without undue reservation.

## Ethics Statement

The studies involving human participants were reviewed and approved by The Research Ethics Committee of Rīga Stradiņš University, Riga, Latvia. The participants provided their written informed consent to participate in this study as well as for the publication of any potentially identifiable images or data included in this article.

## Author Contributions

AP and KM: conception and design of the study. LR-P, AP, and KM: acquisition, analysis, and interpretation of data. AP, KM, and IG: drafting the work and revising it critically for important intellectual content. All authors contributed to the article and approved the submitted version.

### Conflict of Interest

The authors declare that the research was conducted in the absence of any commercial or financial relationships that could be construed as a potential conflict of interest.
